# Fusion of Heterogenous Sensor Data in Border Surveillance

**DOI:** 10.3390/s22197351

**Published:** 2022-09-28

**Authors:** Luis Patino, Michael Hubner, Rachel King, Martin Litzenberger, Laure Roupioz, Kacper Michon, Łukasz Szklarski, Julian Pegoraro, Nikolai Stoianov, James Ferryman

**Affiliations:** 1 Department of Computer Science, University of Reading, Reading RG6 6DH, UK; 2 AIT Austrian Institute of Technology, 1210 Vienna, Austria; 3 ONERA, Département Optique et Techniques Associées (DOTA), Université de Toulouse, 31055 Toulouse, France; 4 ITTI, 61-612 Poznan, Poland; 5 Bulgarian Defence Institute, 1592 Sofia, Bulgaria

**Keywords:** multi sensor fusion, border surveillance, object detection, object tracking, thermal camera, movement sensors

## Abstract

Wide area surveillance has become of critical importance, particularly for border control between countries where vast forested land border areas are to be monitored. In this paper, we address the problem of the automatic detection of activity in forbidden areas, namely forested land border areas. In order to avoid false detections, often triggered in dense vegetation with single sensors such as radar, we present a multi sensor fusion and tracking system using passive infrared detectors in combination with automatic person detection from thermal and visual video camera images. The approach combines weighted maps with a rule engine that associates data from multiple weighted maps. The proposed approach is tested on real data collected by the EU FOLDOUT project in a location representative of a range of forested EU borders. The results show that the proposed approach can eliminate single sensor false detections and enhance accuracy by up to 50%.

## 1. Introduction

Wide area surveillance has become an increasingly important topic with respect to security concerns, not only for large industrial premises and critical infrastructures but for ‘green’ land borders. CCTV installations, based on video- and recently also on thermal-infrared-cameras, are still the backbone of any surveillance solution because they allow fast identification of potential threats and provide good situational awareness for the operator. Because the permanent visual monitoring of a multitude of screens in parallel is nearly impossible for a human operator, automated detection of incursions is the only possible way to scale up to automated wide area green border surveillance.

Automated video detection can be used to reduce the work load of the human operator [1], but it is prone to false detections under low light conditions or adverse weather such as rain or fog. To date, automatic motion detectors are widely used in perimeter security to supplement the camera installations and automatically alert the video operators. Audio detection is sometimes used, especially for the detection and localization of specific strong acoustic events such as glass breaking [2]; the use of specialised microphone hardware has also been reported for source localization [3]. Due to robustness against low light and weather conditions, passive infrared (PIR) detectors [4,5] and radar detectors [6] are frequently applied. PIR devices utilize a pair of pyroelectric sensors to detect heat energy (infrared radiation) in the surrounding environment. A change in signal differential between those two sets off an alarm—PIR can thus detect any IR-emitting object within their visual field of view.

However, even an acceptable false positive rate for a single sensor in any automatic detection system will accumulate a significant number of false alarms as the number of sensors increases in large real world installations. Permanent visual verification of sensors’ false alarms imposes a significant extra workload on the video surveillance personnel and may lead to loss of confidence in the technology. In green border surveillance, natural vegetation plays a crucial role as a source of false detections, as the movement of branches, leaves, or bushes in the wind or rain can, for example, trigger false detections using radar [7]. The wide area surveillance of land borders, with a geographic extension on the order of 100 km, typically situated in dense vegetation, represents an enormous challenge for automated detection technologies [8].

Combining multiple sensor modalities has been a common approach to improve data or detection quality [9]. Sensor systems employ video cameras with automatic person detection and acoustic sensors vested with classification of critical events [10,11]. Data fusion in a rule based fashion for detection of security threats was described in [12]. For accurate positioning of critical events, data fusion of multiple wireless positioning technologies was also investigated [13]. Nevertheless, such systems often follow a direct data fusion approach, meaning that events are triggered by one sensor and are validated by another, different sensor modality [14,15]. This approach works in specific scenarios, but does not exhaust the full potential of data fusion, such as inference based approaches such as [16].

In this paper we present a multi sensor fusion approach using PIR detectors in combination with automatic person detection from thermal and RGB video camera images. The approach adapts the work presented in [17], which combines weighted maps and a linear opinion pool (LOP) to associate data from multiple weighted maps. We show that the fused output can be employed to perform long-term target tracking by calculating the cost of associating fused detections temporally. The performance of the proposed method has been compared with detections of a single sensor in terms of accuracy, precision, and recall values.

## 2. Methodology

### 2.1. Person Detection Data from Video and PIR Sensors

The image frames of thermal and RGB video cameras feed into state-of-the-art detectors ‘You Only Look Once’ (YOLOv5 [18]), which have been trained on the MS COCO (Microsoft Common Objects in Context) dataset [19]. Upon each detection, the corresponding confidence value of the classifier is attributed to the respective grid cells of the density map, as described above. The grid locations have been derived from the camera’s measured field of view (FoV), which represents a triangular shaped area in the weighted map.

The PIR sensor detections were attributed to the surrounding of each PIR sensor’s location. Upon each detection, a defined confidence was attributed to the respective grid cells of the density map on a circular area with 7.5 m diameter around the sensors location on the density map.

### 2.2. Geo-Registration

As each sensor provides data in its own local coordinate systems, a transformation is required to map the detections into a common geodetic coordinate system before they can be fused. A registration procedure is then applied to convert sensors’ raw detections from their respective local coordinate systems to the World Geodetic System (WGS84), chosen as the reference coordinate system in this study. The registered detections are expressed as detection circles or polygons.

The performance of the data fusion step strongly relies on the accuracy of the registration. Therefore, an accurate calibration of each sensor along with a precisely known sensor network geometric configuration are essential to ensure an effective registration. In this study, one of the major challenges lies in the fact that the data to register are highly heterogeneous in terms of intrinsic properties and acquisition methods, leading to a different registration procedure according to two sensors categories: (1) omnidirectional sensors and (2) directional sensors.

#### 2.2.1. Omnidirectional Sensors

For omnidirectional sensors, such as PIR sensors, the procedure is straightforward. The registered detection is centred on the sensor location referenced in the common WGS84 frame and uses the detection range as the radius of the circle element defining the registered detection.

#### 2.2.2. Directional Sensors

For directional sensors, the procedure is more complex. Because the directional sensor’s own local coordinate system is well defined with respect to the WGS84 coordinate system, the Helmert transformation can be calculated. This transformation is a combination of a translation, a rotation, and a scale operation and requires accurate sensor location, height, and position angles (heading, roll, pitch) to register the raw detection. The position of the raw detection in the local coordinate system is provided as a directional vector in polar coordinates (elevation, azimuth), with associated uncertainties used to generate the registered detection as a polygon element. In some cases, the transformation may lead to extreme or illegal registered positions, such as a detection located above the horizon. In those cases, an exception procedure setting the registered detection as the sensor detection FOV based on the sensor FOV and maximum range detection distance is applied. To be processed as an exception, a raw detection must fulfill one of the following conditions:

- The sum of the sensor pitch and detection elevation angles is >= 0 or <−90;
- The distance between the camera and the registered detection is larger than the sensor maximum detection range.

Appropriate sensor calibration and precise knowledge of the location of each sensor within the WGS84 coordinate system is crucial to limit the uncertainty linked to the relative position of the detection. For directional sensors, the accuracy of the registration also strongly relies on the accuracy of the sensor configuration measurements: position angles and sensor height. The rotation and translation matrices applied in the Helmert transform are computed based on those values. Measurement errors are therefore propagated to the registered detection, their impact increasing with sensor height and pitch. A succinct validation based on field GPS data showed satisfying results for this experiment, with registration performance ranging from millimetres to a few tens of metres in the worst case. Those results take into account realistic uncertainties associated with sensor configuration measurements and should be benchmarked against the smartphone GPS accuracy used for validation, which is typically about 5 m under the open sky.

### 2.3. Multi Sensor Fusion with Weighted Maps

Multi sensor data fusion is widely used for location based applications, including sensor networks. The potential allows for use of such methodologies in different sectors, such as border surveillance, surveillance of critical infrastructure, as well as in the automotive sector. To estimate an object’s location at a specific time, common approaches are feature methods or location based [20]. For example, in the automotive sector, a relatively small sensor network is used to model the vicinity of a vehicle and derive appropriate actions using a feature based approach [21]. Nevertheless, the scalability of such methods is questionable in the context of surveillance tasks that include a large amount of sensors (e.g., in the order of 1000 for a land border) and a very large geographical extent, as is the case in border surveillance. Location based maps, where each cell represents the probability of an event, is therefore a more suitable approach to reduce the computational cost and meet the real-time capability that is still essential in both sectors. Occupancy grid maps [22] and density maps [23] are common solutions for location based maps.

In this work, we adapt the approach presented in [17], in which the concept of weighted maps was introduced. The weighted maps concept is derived from probabilistic occupancy maps [24]. Essentially, the fusion process is modeled by a two step approach. First, the update process of weighted maps is introduced. This models the spatio-temporal behaviour of a weighted map inferred with events reported by a sensor or even a set of sensors of the same sensor modality. The second step is the fusion of multiple weighted maps. A linear opinion pool (LOP) [25] was used for this step. One main advantage of a LOP is that sensors yielding highly reliable data can be prioritized by increasing the weights employed in the LOP. This also allows to fuse sensor data in a rule based fashion, which helps to interpret the fusion methodology more easily as well as easing the parameterization. For completeness, a summary regarding the core methodology of the work presented in [17] is provided in this section.

According to [17], a weighted map $M^{i}$ is defined on a grid *G* that represents the area of interest of a sensor $S_{i}$ that is able to report or give evidence about certain events in its vicinity, such as the PIR sensors as well as the visual camera and the thermal camera. Here, $m_{j,k}^{i}$ are the weights modeling the time dynamic behaviour of the weighted map for each cell (j,k)∈G. In this work, all of the sensors $S_{i}$ provide localized events reported at a specific time $t_{E}$. Additionally, each sensor is able to estimate its confidence regarding the reported event. For example, a score of the classification whilst detecting a person, or one or zero in case of a binary classification. Note that in this work we use this confidence as weights ${\left\{w_{E,j,k}\right\}}_{(j,k)\in G}$ as described in [17]. At the time of occurrence $t_{E}$ of an event reported by a sensor $S_{i}$, we can calculate the state (weight) of the current cell (j,k)∈G according to:

(1) $$m_{j,k}^{i}\left(t_{E}\right)=\min\left(w_{max},m_{j,k}^{i}\left(t_{0}\right)e^{-\lambda_{i}\left(t_{E}-t_{0}\right)}+w_{E,j,k}\right).$$

Thus, Equation (1) describes the update process, modeling the spatio-temporal behaviour of a weighted map. To reduce the weights provided by older events, an exponential decay with a decay constant $\lambda_{i}$ was introduced. For a detailed explanation on how the update process is done, please refer to [17]. In our setup, a typical example for a thermal sensor would be λ=0.5 s, $w_{E}=\frac{1}{30}$, and $w_{max}=1$. Here, we assume that the thermal sensor yields approximately 30 events per second, in case an event is present and detected. Using this approach, we see that the weighted maps $M^{i}$ yield high weight values if the corresponding sensors provide a large number of events at the same location in a short timespan.

**Figure 1:**
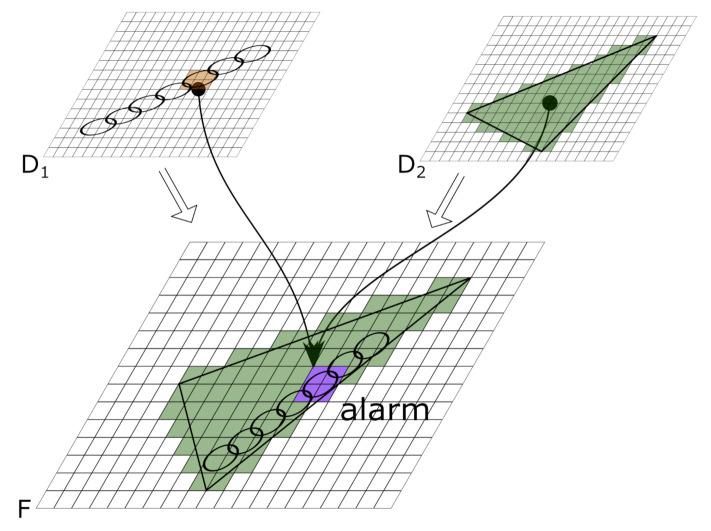
Illustration of fusion approach with two weighted maps. Weight map $D_{1}$ for PIR detections (**left**). Weight map $D_{2}$ for detections of person classification using thermal camera images (**right**). Resulting alarm of fusion (**bottom**).

In the second step, the fusion of the weighted maps, the LOP is employed as described in [25]. In this work, we chose the normalization factor $\alpha =\omega_{max}$. Thus, the fusion process is modeled by evaluating the LOP $F_{j,k}\left(t\right)$ of different weighted maps $M^{i}$ for each weight $m_{j,k}^{i}\left(t\right)$ at any point in time *t*. In Figure 1, the fusion process is depicted using a weighted map $D_{1}$ for PIR detections (left) and a weighted map $D_{2}$ for detections of person classification employed on thermal images (right). Finally, a decision can be made to trigger an alarm if a certain threshold τ∈[0,1] is exceeded for each individual cell (j,k)∈G. The alarm resulting from the decision process is localized at those cells of the grid where the threshold τ is exceeded. This set of cells is denoted as $\left\{\left(j,k\right)\in G:F_{j,k}\left(t\right)>\tau \right\}$. An example of a triggered alarm and its location is shown in Figure 1 in purple (bottom).

Note that in this paper we do not include the restricted fusion map as described in [17]. In this way, we also allow the triggering of alarms in areas where no overlap of sensors of different types occur in the case where sufficient confidence is provided. This results in higher coverage of the area of interest. Specifically, in the task of detection through foliage, this approach turned out to be more suitable. An example (illustrated by Figure 1) of the expression using a weighted map $D_{1}$ for PIR detections and a second weighted map $D_{2}$ for detections of a person in thermal videos can be written as:

(2) $$F\left(t\right)=\frac{1}{2}D_{1}\left(t\right)+\frac{1}{2}D_{2}\left(t\right)>\tau .$$

In this example, the weights are chosen as $\omega_{1}=\omega_{2}=\frac{1}{2}$. In our work, the weights were chosen uniformly, i.e., $\omega_{i}=\frac{1}{3}$ for fusion of three sensors modalities and $\omega_{i}=\frac{1}{2}$ for fusion of two sensor modalities. The threshold for triggering an alarm was chosen to be τ=0.8. Generally, in this methodology, the parameter τ is use to parameterize the sensitivity of the fusion process. The higher τ is chosen, the more sufficient evidence the sensors (i.e., the weighted maps) need to provide overtime to be very confident in the decision. This parameter typically is chosen empirically based on the knowledge of sensors behaviour and the required sensitivity.

### 2.4. Tracking of Fused Objects

Within a surveillance system, a natural application of the fused data would be to feed a tracking system that would allow, in the foreseen application, following the movement of an person illegally crossing a border. With this application as a target, we have developed a simple tracker to analyse the potential use of fused data on tracking.

The tracking system works by building a model of the object exclusively based on its position and time stamp. At the first object detection, the model is initialised with the position and timestamp of that detection. A track model is defined thus as the following tuple:

(3) $$T_{i}=\left\{x_{i},y_{i},t_{i}\right\}$$

where x,y, and *t* correspond, respectively, to the latitude, longitude, and timestamp of the point. If several object detections occur at the same time, there are as many model templates created as there are detections simultaneously received. Subsequent detections are added to a given track model depending on the cost involved on appending the detection to the track. The cost is defined as the distance between the incoming detection and the track candidate.

Let $d_{s}\left(T_{i},o\right)$ be the spatial distance between the most recent point in the track $T_{i}$ and the incoming detection *o*. The spatial distance is calculated as the Euclidean distance between the latitude and longitude of the two points.

Let $d_{t}\left(T_{i},o\right)$ be the temporal distance between the most recent point in the track $T_{i}$ and the incoming detection *o* given by the substraction of the two point timestamps. The cost of appending object *o* to track $T_{i}$ will be then calculated as:

(4) $$C=2-e^{-{\left(d_{s}*\tau_{s}\right)}^{2}}-e^{-{\left(d_{t}*\tau_{t}\right)}^{2}}$$

where $\tau_{s}$ and $\tau_{t}$ are, respectively, spatial and temporal similarity parameters tuned empirically for our current implementation.

The object is appended to the track if the cost is less or equal than a given threshold $\tau_{c}$; otherwise that object will initialise another track. In case of multiple incoming detections and multiple track candidates, a Hungarian algorithm [26] was implemented so that the associations between detections and tracks incurs the minimum cost.

### 2.5. Data Description

Data were collected at a simulated border site where actors were asked to simulate typical border scenarios under realistic conditions, i.e., different times of the day and the weather conditions prevailing on the day. Prior to the collection of data, an ethics approval process was adopted. The actors were provided an information sheet describing the purpose of the study, the data that would be collected, and how it would be stored and used. Written informed consent to take part in this study was obtained for all participants.

**Figure 2:**
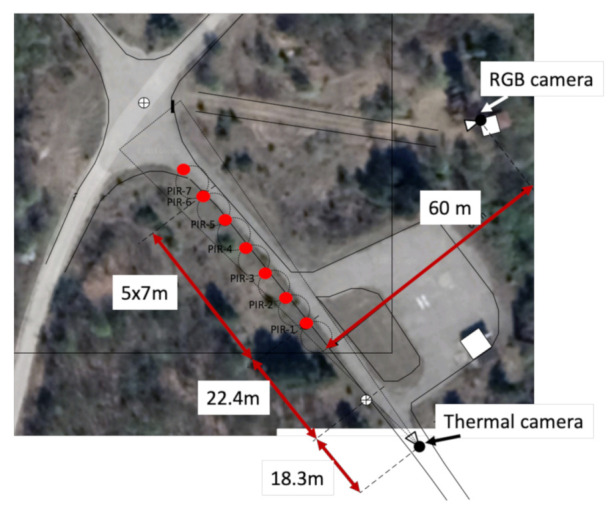
Sensor deployment in the experimental setup. RGB and thermal cameras are signed with arrows. PIR sensors indicated with red circles.

**Figure 3:**
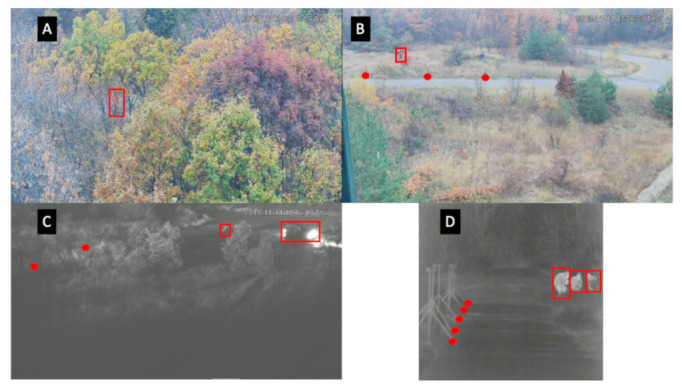
Examples of images contained in the dataset. Detected objects in these images are enclosed in a red bounding box. Deployed PIR sensors are marked with red circles. (**A**) A person is observed through the trees on the RGB camera; (**B**) a person is observed near the road on the RGB camera; (**C**) a person and a vehicle are observed by night on the RGB camera; (**D**) three people are observed hiding through the trees on the thermal camera.

The simulated border site was characterised by a road with a car park. A simulated border was established down one side of the road, as shown in Figure 2, and both sides of the road had areas of foliage. Two types of sensors were deployed to monitor this area: PIR sensors and video cameras (one thermal and one RGB camera). Whereas Figure 2 gives an overview of the sensor placement as well as the dimensions of the area, some sample images captured by the thermal and RGB cameras are given in Figure 3. These images are representative of the challenge addressed in this work, namely, the though-foliage detection of people in green areas. The problem is fragmented occlusion, which appears in the case of through-foliage detection in natural (green) environments. This detection is important and very much needed by border authorities worldwide for enhancing border security operations on green borders. Fragmented occlusion usually appears simultaneously as partial or full occlusions, which undoubtedly affect the performance of automatic people detectors. From Figure 3 it can be observed how challenging the person detection is, taking into account the foliated environment and natural conditions that can be particularly difficult, such as low light during the night. Fragmented occlusion has become a hot topic in automated surveillance at green borders where through-foliage detection is key. The collected dataset has, in part, been made available to the scientific community to foster developments in this new area [27] and aims to fill the gap currently existing on datasets addressing through-foliage detection.

Six PIR sensors were deployed along the simulated border at 7 m intervals. Each PIR sensor has a range of approximately 10 m and a FoV of 90° parallel to the simulated border. The thermal camera was deployed approximately 50 m from the PIR sensors on the simulated border, with the FoV parallel to the simulated border. The thermal camera used was the FLIR F-606E. As this camera detects heat, it is possible to detect a person even in poor weather and lighting conditions, making it an ideal complimentary sensor for this application. This camera has a thermal spectral range of 7.5 μm to 13.5 μm, and a FoV of 6.2° × 5° [28]. The RGB camera used was the DH-SD6AL830V-HNI 4K Laser Pan-Tilt-Zoom (PTZ) Network Camera. It features 12Megapixel STARVIS™ CMOS, powerful optical zoom (×30), and accurate pan/tilt/zoom performance; this camera provides an all-in-one solution for capturing long distance video surveillance for outdoor applications. The sensor layout can be seen in Figure 2.

**Figure 4:**
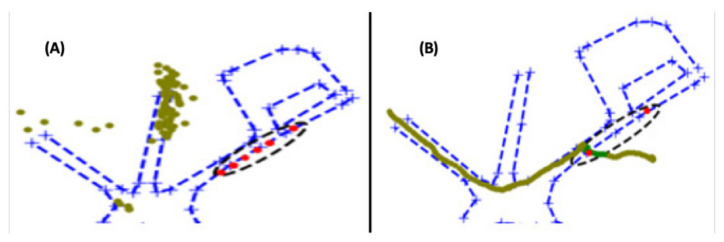
ZoI depicted with a black dashed line for performance evaluation with data from two tested scenarios: (**A**) activity happening outside the ZoI; (**B**) one person crossing the ZoI. PIR detections are shown in red (particularly in panel (**A**), note the appearance of PIR false alarms); GT data not included in the analysis in light green; GT data included in the analysis in dark green.

In addition to the PIR sensors and the RGB and thermal cameras, participants each carried a mobile phone with GPS capability. An app called ‘GPS Logger for Android’ [29] was installed on each of these devices and used to record each actor’s location during the data collection to be used as ground-truth (GT) data. A summary of all characteristics of the dataset addressed in this paper are given in Table 1. The area that is within range of the PIR and thermal sensors is referred to as the zone of interest (ZoI) and is illustrated in Figure 4.

Participants were asked to simulate typical border scenarios based on a range of predefined scripts. These scripts include activities such as: a single actor, or group of actors, simulating a border crossing though the ZoI and negotiating the surrounding area; a single actor, or group of actors, simulating a border crossing though the ZoI and waiting to be picked up by a vehicle; or actors performing simulated illicit activities, such as a vehicle loading or unloading illicit material near the border but not necessarily in the ZoI. One of the objectives of the data collection was to perform it under different representative conditions to evaluate how effective the system would be to detect the activities. As such, two different behaviour modalities were scripted. The first mode was naïve behaviour, where the actor simulates being unaware of the surveillance system and performs the activity without hiding from cameras or PIR sensors; in the second mode, system aware behaviour mode, the actor would act in such a way to know at least the existence of a surveillance system and try to move more quickly and silently and attempt to partially hide. From the data collected, fifteen sequences were generated representing simultaneous recording by RBG, thermal, and PIR sensors. The selection of sensors was considered appropriate for analysis to demonstrate the reduction in false detections using the fusion techniques described in this paper. A summary of these sequences is given in Table 2.

### 2.6. Evaluation Methodology

**Figure 5:**
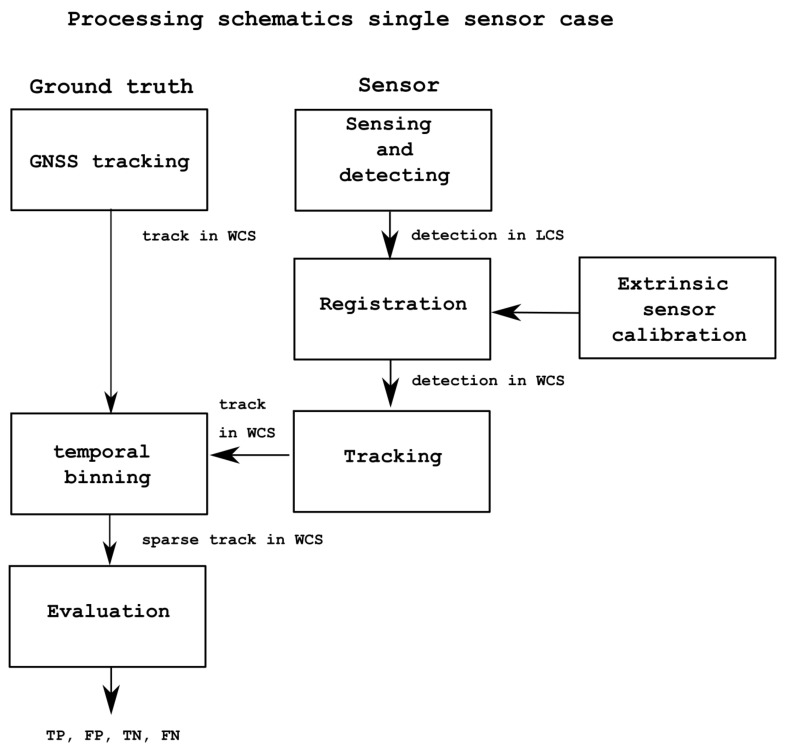
Schematic of data processing for single sensors (e.g., PIR and thermal camera) from detection to transformation between local coordinate system (LCS) to world coordinate system (WCS) and evaluation.

**Figure 6:**
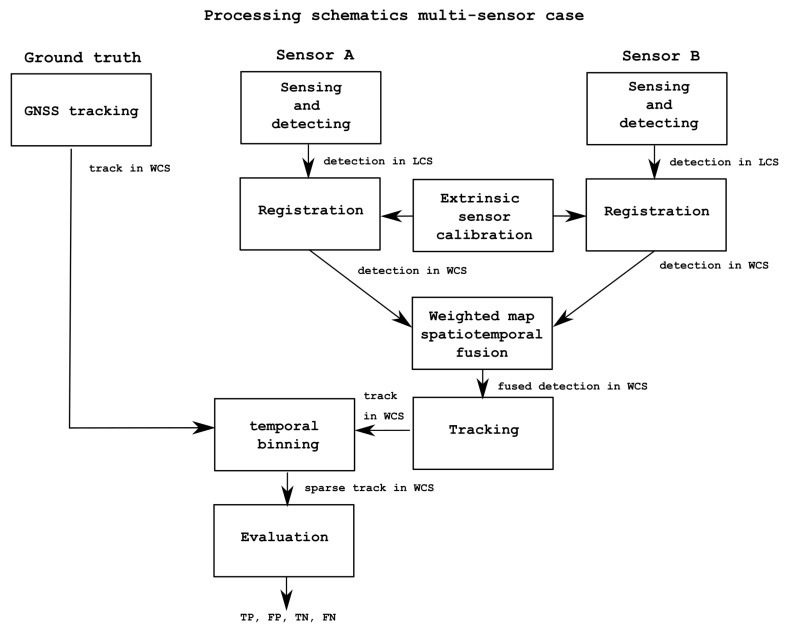
Schematic of data processing for multi fusion approach from detection to fusion to transformation between local coordinate system (LCS) to world coordinate system (WCS) and evaluation.

In order to test how the approach described in this paper can reduce the false alarm rate of a single sensor’s performance, we compare the object detection performance of individual detectors and that of fused output of combined detectors taking as ground-truth the GPS data collected from the actors’ phones. We evaluate the proposed multi sensor fusion approach on the area where the FoV of the thermal and RGB cameras overlap the detection range of the PIR sensors. The full processing schematic for single sensor evaluation is summarized in Figure 5; the fused output evaluation is given in Figure 6. Note that the evaluation process includes a tracking component. The influence of the tracking component alone in the proposed approach is evaluated by inputting into the system the ground-truth data themselves as detection data only (no knowledge of tracking ID) and allowing the tracker to associate the individual detections, form the tracks, and assign an ID. The resulting tracks are compared with the GT. These results are discussed in the next section, but it is expected that the tracker would have almost 100% accuracy when the input data are the ground-truth themselves; this would confirm the tracking component does not distort or influence the results from the fusion algorithm in our proposed approach.

We thus concentrated the evaluation on a zone of interest inside the FoV of the thermal and RGB cameras and also covering the PIR sensors (see Figure 2 and Figure 4). Note that the evaluation focuses on establishing whether the activity in the ZoI is true or false when compared to GT data, which are given by the GPS sensors carried by actors performing activities (or not) in the ZoI. The full processing schematic for the multi fusion approach evaluation is summarized in Figure 6.

The test data comprises two types:

- The activity is outside the ZoI so that the full potential of the fusion approach on filtering false detections can be evaluated (see Figure 4A).
- The activity happens inside the ZoI so that the accuracy of detections can be evaluated (see Figure 4B).

To solve for different sampling frequencies, all data were analysed in temporal windows of 1 s duration. Detection data and GT data were compared inside these temporal windows with typical receiver operator characteristic performance measures of true positives (TP), false positives (FP), true negatives (TN), and false negatives (FN) defined as follows:

- True positive (TP): In a given temporal window, a system detection and a GT object exist inside the ZoI.
- False positive (FP): In a given temporal window, a system detection exists inside the ZoI but no GT object is found.
- True negative (TN): In a given temporal window, no system detection exists inside the ZoI and no GT object is found.
- False negative (FN): In a given temporal window, no system detection exists inside the ZoI, however, a GT object is found.

Typical performance measures of accuracy, precision, and recall can then be calculated:

(5) $$Accuracy=\frac{\left(TP+TN\right)}{\left(TP+TN+FP+FN\right)}$$

(6) $$Precision=\frac{TP}{\left(TP+FP\right)}$$

(7) $$Recall=\frac{TP}{\left(TP+FN\right)}$$

## 3. Results and Discussion

Fifteen sequences (scenario scripts) have been evaluated to assess the proposed approach. Data were analysed in time intervals of 1 s for all sequences. First, as tracking is employed in the evaluation process of single sensors (see Figure 5) and multiple sensor fusion (see Figure 6), the influence of the tracking component alone in the proposed approach is evaluated by inputting into the system the ground-truth data itself as detection data only (no knowledge of tracking ID) and allowing the tracker to associate the individual detections, form the tracks, and assign an ID. The resulting tracks are compared with the GT. These results are shown in Table 3. It can be observed from the results that the matching with the GT is almost perfect except for a few cases. Accuracy, precision, and recall are at 100% except for three cases (scripts D, H, and M). In these scripts, the lowest accuracy value is 91%, and precision is always at 100%, except for one case at 98%. Only the recall has some lower values on the aforementioned three scripts, ranging from 71% to 91%, and 100% otherwise. This means that the tracker component commits some mistakes in generating the tracks, making the recall drop by 20% ion average across these three scripts; these tracking mistakes are limited, taking into account that overall only three scripts out of fifteen result in results that are not matching perfectly with the GT. Examining these scripts in Table 2, they correspond to group activities where there are small to large groups (3 to 7 people) moving together in the area. It is well known in tracking that following different targets close to each other can be challenging and produce errors in tracking. It also must be taken into account that the GPS data are also sensor data and contain some irregular sampling; this produces even more challenges to the tracking component, which overall can be deemed to work considerably well.

Second, the evaluation was further performed, first taking one single sensor at a time, and then the different possible sensor combinations for data fusion. Each line in Table 4 shows the resulting evaluation for single sensors and for the different fusion combinations of sensors.

A closer look at Table 4 shows that the usage of this approach when combining all sensors yields one of the lowest FP values. The thermal camera has the lowest FN value but also the highest FP value, meaning that the sensor is very sensitive but at the same time is producing a considerable number of false detections. The PIR sensor in itself has the lowest FP value, but, at the same time, the number of TP is also the lowest. The consequence of this is that the proposed approach shows a significant reduction in FPs (between 95% reduction when compared to RGB and 91% when compared to the thermal camera sensor).

It must be noted that PIR sensors show a compromise between not producing FPs due to movement of tree branches and leaves and being sensitive enough to detect a person passing by. The PIR sensor sensitivity was moderate, which produced, in consequence, the lowest value of FPs but at the same time has the lowest value of TPs and the highest value of TNs. This translates to the PIR sensor having a good precision but the lowest recall among individual or fusion combined sensors. In contrast, the RGB and thermal camera sensors appear to have an opposite operation; their sensitivity is high and therefore their recall values are the highest in Table 4; however, their precision is also the lowest, and this can be seen by the high number of FPs they are producing. Having a highly sensitive system is generally preferred in security systems, although the downside is generating a number of false detections that must be verified by a security guard: therefore the importance of our fusion approach in filtering false detections.

Table 4 shows that the proposed approach actually balances the two operation modes between PIR and camera (RGB, thermal) sensors. The best results are achieved when all sensor data are fused. The combination of PIR-RGB-thermal sensors leads to the best accuracy and precision values, 0.88 and 0.55, respectively. The recall is, however, not as large (0.34) given that the PIR sensor has minimal recall in itself, and this influences the overall fusion results. The tracking component may also have an influence as shown before, and recall values could potentially rise by about 20%, bringing recall values potentially to 41%.

Table 5 details the performance of individual sensors and the different sensor fusion results according to the number of people appearing in the scene (single person, small group of three people, or large group of ten people) and also according to the different behaviours adopted (naïve person or system aware). In general, it can be observed that the naïve behaviour allows the system to produce a significant higher number of true positives. In system aware, the actors perform realistic movements attempting to hide from the system sensors, and fewer TP detections are achieved. The values of precision and recall decrease, particularly in the case of one person on the scene or a large group of ten people. Interestingly, the performance seems not to be affected when monitoring small groups of three people. This can be due to the fact that, within the small group, the system continuously detects one of the members, whereas if it is a single person, it is much more difficult to continuously track that person, and if it is a large group of people, it is more difficult to detect all members. It is interesting to observe that the same patterns noted before for Table 4 can also be observed in Table 5; namely, RGB and thermal cameras are sensitively tuned and have a good recall but also produce a significant number of false positives. PIR sensors have a lower recall but a better precision. The fusion of sensors enhances the overall precision at the expense of the recall for some individual sensors. Different sensor combinations produce best results according to the number of people in the scene and the actors’ behaviour; however, it can be seen that combining all sensor data always leads to the best or second-best results for detection.

The results obtained with the proposed approach are very encouraging given the fact that the actors crossed the simulated border quickly and then either hid or continued their path either on the road or across the foliage on the other side of the road. Sharp movements and foliage represent a significant detection challenge. Camera sensors are sensitive enough to detect people as far as possible, even through foliage, although with the downside of generating false positives. Notwithstanding this, our proposed approach manages to filter most FPs. Regarding true positives, these would certainly be improved by adding more sensors into the fusion system, giving a better coverage of the area targeted for surveillance; some possibilities include adding crossing cameras, more PIR sensors, or other types of sensors such as seismic or airborne sensors.

Nevertheless, there are drawbacks of the proposed methodology that have been uncovered during this work. First of all, we would like to state that the proposed fusion methodology is able to satisfactorily balance out the drawbacks and strengths of the employed sensors as described previously. However, the proposed fusion method heavily relies on the quality of the input data. Particularly, in the case of through-foliage detection, it is quite challenging to reliably detect the desired event for each individual sensor modality. Consequently, this transfers to the output of the fusion, which especially can be seen in the recall in Table 4. We would like to investigate this trade-off more in future work using supplementary sensor modalities.

Additionally, it was hard to find open source datasets to compare our work to. We did not find any dataset that was directly comparable to the one collected in this work. The reason for this is mainly due to the lack of contributions in the field of through-foliage applications with the dependencies of the defined scenarios within this work. Indeed, we would like to contribute to this field in the future.

Finally, we also would like to acknowledge that parameterization of the fusion methodology can be challenging with increasing numbers of sensor modalities. Obviously, in a sensor network, it is desired to use as many sensor modalities as possible to increase the probability of a sensitive system with high accuracy and recall so as to reduce the number of false alarms generated. However, simply fusing all the sensors together in a uniform way will not produce the best results. For this reason, most of the time sensors with complementary attributes are fused (e.g., PIR: high accuracy and precision; cameras: good recall). As a result, a significant amount of domain know-how as well as understanding of the sensor models is necessary to fully exhaust the potential of the proposed methodology. In the future, we would like to focus our research on how to reduce the complexity of parameterization with the overall goal to increase the robustness and therefore the reliability of such a system. One example is to use neural networks or deep learning approaches to automatically learn the weights and threshold in the decision process [17] based on the employed sensor modalities and use-cases.

**Figure 7:**
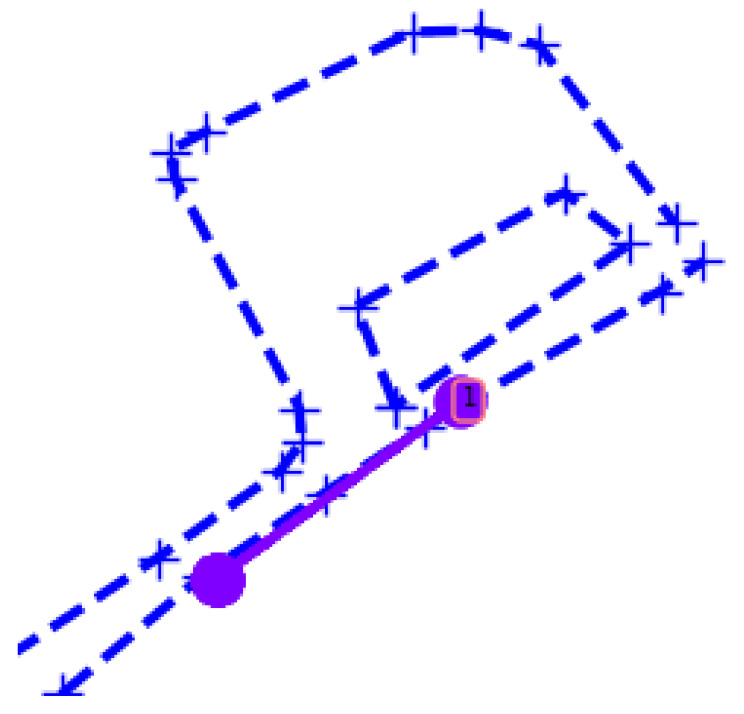
Tracking from fusion output. A person walks along the road; their presence is confirmed by the firing of PIR sensors and the thermal camera. The tracking system attributes an ordered tracking ID, ‘1’, for this tracked object.

Regarding our tracking system, Figure 7 shows, as an application example, the track resulting from the fused data of Seq. E in Table 2. The tracking is coherent given the activity, and its application for monitoring the area is promising given that the fusion ‘cleans’ most false detections, which certainly would perturb the tracking, either making the track bounce to false positions, causing tracks to change IDs, or breaking tracks and create new ones, provoking fragmentation. All of these are well known issues when tracking is corrupted with noisy data.

## 4. Conclusions

In this paper, we presented a multi sensor fusion and tracking system using passive infrared detectors in combination with automatic person detection from thermal and visual video camera images. We adopted a weighted map based fusion approach combined with a rule engine. The geo-reference detections of PIR, RGB, and thermal detections resulting from an open source video classification software were used as input for the weighted maps.

We evaluated the fusion using fifteen different sequences corresponding to different acting scripts and compared the results of single sensor detections and the weighted map based fusion approach. We conclude the following results:

- A significant reduction in FPs, which also translates in an increased number of TNs;
- An increase in the accuracy (28% increase compared to RGB and 47% compared to thermal);
- An increase in the precision (more than 220% increase compared to RGB and 71% compared to thermal);
- Larger groups of people, and people behaving in a naive way, allow for collecting more detections, which in turn facilitates delivery of alerts. The increased number of FP from individual sensors is well managed in the fusion.

The fusion system proves to be effective for border surveillance tasks, but its effectiveness is dependent on the sensors delivering the input to the fusion itself. With the presented fusion approach, we achieved a significant reduction in false alarms that were mostly due to adverse weather conditions and foliage producing false detections in the deployed sensors. However, true detections will only be confirmed if there is sufficient evidence from different sensors to assert the event; thus, sensors themselves must comply with a minimum level of accuracy on their own. It is noteworthy that the proposed fusion approach can work with any number of added sensors. Indeed, in the future we would like to experiment with a larger set of sensors including seismic, Pan-Tilt-Zoom (PTZ) cameras, and airborne sensors. This paper represents an experimental exploration where the focus is to cover areas of high interest (illegal crossings at borders) from a border guard perspective; employing sensors offering broader area coverage is also part of our future studies. Data fused from heterogenous sensors can feed different components in a surveillance system. In this paper, we show the applicability of a tracking system on fused data as a promising application. Overall, our current results show an important step for the use-case of through-foliage detection with multi sensor fusion.

## Figures and Tables

**Table 1:** Dataset specifications.

Device	Model or Make	Sequences Recorded	Data Recorded	Recording Resolution
Thermal camera	FLIR F-606E	15	10,608 frames	4 fps
RGB camera	Dahua DH-SD6AL830V-HNI 4K PTZ Network Camera	15	7956 frames	3 fps
PIR sensor	Custom made	15	44.2 min	0.5 Hz
GPS tracker	GPS Logger app on Samsung phone	15	44.2 min	3 Hz

**Table 2:** Characteristics of the sequences used for analysis.

Seq.	Local Time	Duration	Behaviour	Number of Actors	Activity Description
A	13/11/2019 16:36:17	133 s	Syst. Aware	3	A group of three actors simulate crossing the simulated border in the ZoI then walk along the road. The group splits and continue walking in different directions.
B	13/11/2019 16:42:59	157 s	Naïve	3	A group of two actors simulate crossing the simulated border in the ZoI then wait near the road. A car arrives shortly afterwards and someone exits the car to fetch the two people.
C	13/11/2019 16:51:14	224 s	Naïve	10	A large group of seven actors simulate crossing the simulated border in the ZoI to meet three other actors, then walk along the road.
D	13/11/2019 17:03:52	116 s	Naïve	3	A group of three actors simulate crossing the simulated border in the ZoI then walk along the road. The group splits and continue walking in different directions.
E	14/11/2019 13:23:54	117 s	Syst. Aware	10	A large group of seven actors simulate crossing the simulated border quickly and silently in the ZoI to meet three other actors, then walk quickly along the road.
F	14/11/2019 15:22:47	192 s	Naïve	3	A group of three actors simulate crossing the simulated border in the ZoI then walk along the road. The group splits and continue walking in different directions.
G	14/11/2019 15:30:44	263 s	Naïve	1	An actor simulates crossing the simulated border in the ZoI then walks along the road.
H	14/11/2019 15:44:15	160 s	Naïve	10	A large group of seven actors simulate crossing the simulated border in the ZoI to meet three other actors, then walk along the road.
I	14/11/2019 15:59:56	171 s	Syst. Aware	1	An actor simulates crossing the simulated border in the ZoI then walks along the road.
J	14/11/2019 16:06:37	112 s	Syst. Aware	3	A group of two actors simulate crossing the simulated border in the ZoI then hide near the road. A car arrives shortly afterwards and someone exits the car to fetch the two people.
K	14/11/2019 16:11:28	104 s	Syst. Aware	3	A group of three actors simulate crossing the simulated border in the ZoI then walk along the road. The group splits and continue walking in different directions.
L	14/11/2019 16:17:24	201 s	Naïve	3	A group of two actors simulate crossing the simulated border in the ZoI then wait near the road. A car arrives shortly afterwards and someone exits the car to fetch the two people.
M	14/11/2019 17:00:44	256 s	Naïve	10	A large group of seven actors simulate crossing the simulated border in the ZoI to meet three other actors, then walk to an open area.
N	14/11/2019 17:11:12	167 s	Naïve	3	A group of three actors simulate crossing the simulated border in the ZoI then walk along the road. The group splits and continue walking in different directions.
O	14/11/2019 17:24:23	279 s	Naïve	1	An actor simulates crossing the simulated border in the ZoI then walks to an open area.

**Table 3:** Tracker evaluation with GPS-GT data.

Script	TP	FP	TN	FN	Accuracy	Precision	Recall (TPR)
A	14	0	120	0	1	1	1
B	38	0	123	0	1	1	1
C	69	0	165	0	1	1	1
D	12	0	105	5	0.96	1	0.71
E	3	0	115	0	1	1	1
F	1	0	192	0	1	1	1
G	20	0	244	0	1	1	1
H	71	0	95	16	0.91	1	0.82
I	10	0	162	0	1	1	1
J	4	0	109	0	1	1	1
K	6	0	99	0	1	1	1
L	4	0	198	0	1	1	1
M	63	1	202	6	0.97	0.98	0.91
N	4	0	164	0	1	1	1
O	9	0	271	0	1	1	1

**Table 4:** Comparison of confusion matrices using single sensor detections and fusion of combined sensor detections for all sequences.

Sensor	Sequences Evaluated	TP	FP	TN	FN	Accuracy	Precision	Recall (TPR)
PIR	15	60	54	2321	295	0.87	0.53	0.17
RGB	15	241	1878	2153	114	0.55	0.11	0.68
Thermal	15	270	1056	1308	85	0.58	0.20	0.76
Fusion-RGB-PIR	15	27	18	2347	328	0.87	0.60	0.08
Fusion-Thermal-PIR	15	175	267	2097	180	0.84	0.40	0.49
Fusion-Thermal-RGB	15	150	174	2193	205	0.86	0.46	0.42
Fusion-Thermal-RGB-PIR	15	122	98	2267	233	0.88	0.55	0.34

**Table 5:** Comparison of detections from single and fused sensors according to different group sizes and behaviour. The best system performance by category is highlighted in green and the second best performance is highlighted in yellow.

One Person in Acted Scripts																
	Naïve Behaviour								System Aware							
Sensor	Squences Evaluated	TP	FP	TN	FN	Accuracy	Precision	Recall	Sequences Evaluated	TP	FP	TN	FN	Accuracy	Precision	Recall
PIR	2	1	0	515	28	0.947944	0	0.025	1	0	2	160	10	0.93023256	0	0
RGB	2	1	19	502	28	0.914103	0.055556	0.025	1	0	3	159	10	0.9244186	0	0
Thermal	2	14	261	254	15	0.495346	0.050821	0.625	1	8	58	104	2	0.65116279	0.12121212	0.8
Fusion-RGB-PIR	2	0	1	514	29	0.944156	−0.5	0	1	0	1	161	10	0.93604651	0	0
Fusion-Thermal-PIR	2	4	27	488	25	0.904437	0.153846	0.1	1	3	9	153	7	0.90697674	0.25	0.3
Fusion-Thermal-RGB	2	4	5	510	25	0.944372	0.285714	0.1	1	0	6	156	10	0.90697674	0	0
Fusion-Thermal-RGB-PIR	2	3	5	510	26	0.942478	0.25	0.075	1	0	5	157	10	0.9127907	0	0
Group of Three People in Acted Scripts																
	Naïve Behaviour								System Aware							
Sensor	Sequences Evaluated	TP	FP	TN	FN	Accuracy	Precision	Recall	Sequences Evaluated	TP	FP	TN	FN	Accuracy	Precision	Recall
PIR	3	2	3	459	20	0.9432647	0.22222222	0.0392157	2	7	5	216	13	0.92622549	0.55714286	0.3452381
RGB	3	14	150	438	8	0.7373253	0.05833333	0.2745098	2	13	60	217	7	0.79744613	0.21912833	0.6547619
Thermal	3	14	173	288	8	0.64039017	0.11438596	0.5294118	2	14	35	184	6	0.81506752	0.37310606	0.69047619
Fusion-RGB-PIR	3	2	3	458	20	0.94216117	0.33333333	0.1029412	2	3	1	218	17	0.92562189	0.5	0.10714286
Fusion-Thermal-PIR	3	10	45	416	12	0.87308514	0.17272727	0.3872549	2	11	18	201	9	0.88070362	0.40865385	0.48809524
Fusion-Thermal-RGB	3	9	21	441	13	0.92494719	0.24679487	0.2401961	2	12	6	213	8	0.94157783	0.625	0.57142857
Fusion-Thermal-RGB-PIR	3	8	14	448	14	0.93680682	0.28888889	0.2205882	2	11	7	212	9	0.93102345	0.55194805	0.48809524
Group of Ten People in Acted Scripts																
	Naïve Behaviour								System Aware							
Sensor	Sequences Evaluated	TP	FP	TN	FN	Accuracy	Precision	Recall	Sequences Evaluated	TP	FP	TN	FN	Accuracy	Precision	Recall
PIR	3	43	36	434	182	0.67484461	0.53030639	0.182742	1	1	4	111	2	0.94915254	0.2	0.33333333
RGB	3	180	1459	354	45	0.27734992	0.11761628	0.7966017	1	2	62	103	1	0.625	0.03125	0.66666667
Thermal	3	180	215	247	45	0.60355232	0.45801282	0.8035982	1	2	28	87	1	0.75423729	0.06666667	0.66666667
Fusion-RGB-PIR	3	19	9	454	206	0.67420159	0.62698413	0.0837914	1	0	1	114	3	0.96610169	0	0
Fusion-Thermal-PIR	3	132	100	362	93	0.70501256	0.57446461	0.5827087	1	2	22	93	1	0.80508475	0.08333333	0.66666667
Fusion-Thermal-RGB	3	101	77	386	124	0.69969087	0.58468281	0.4439447	1	2	12	103	1	0.88983051	0.14285714	0.66666667
Fusion-Thermal-RGB-PIR	3	86	43	419	139	0.72977651	0.64847884	0.3694819	1	2	11	104	1	0.89830508	0.15384615	0.66666667

## Data Availability

The data that support the findings of this study are part of the research project and are not publicly available.
